# Emergent approaches to the meta-analysis of multiple heterogeneous complex interventions

**DOI:** 10.1186/s12874-015-0040-z

**Published:** 2015-06-02

**Authors:** G. J. Melendez-Torres, Chris Bonell, James Thomas

**Affiliations:** Centre for Evidence-Based Intervention, Department of Social Policy and Intervention, University of Oxford, Oxford, UK; Social Science Research Unit, UCL Institute of Education, University College London, University of London, London, UK

**Keywords:** Systematic review, Complex interventions, Meta-analysis, Multiple interventions meta-analysis, Network meta-analysis

## Abstract

**Background:**

Multiple interventions meta-analysis has been recommended in the methodological literature as a tool for evidence synthesis when a heterogeneous set of interventions is included in the same review—and, more recently, when a heterogeneous set of complex interventions is included. However, there is little guidance on the use of this method with complex interventions. This article suggests two approaches to model complexity and heterogeneity through this method.

**Discussion:**

‘Clinically meaningful units’ groups interventions by modality or similar theory of change, whereas ‘components and dismantling’ separates out interventions into combinations of components and either groups interventions by the combination of components they demonstrate or extracts effects for each identified component and, possibly, interactions between components. Future work in systematic review methodology should aim to understand how to develop taxonomies of components or theories of change that are internally relevant to the studies in these multiple interventions meta-analyses.

**Summary:**

Despite little meaningful prior guidance to its use in this context, multiple interventions meta-analysis has the potential to be a useful tool for synthesising heterogeneous sets of complex interventions. Researchers should choose an approach in accordance with their specific aims in their systematic review.

## Background

Multiple interventions meta-analysis has been recommended in the methodological literature as a tool for evidence synthesis when a heterogeneous set of interventions is included in the same systematic review. This meta-analytic method generates an effect estimate for each and every pairing of intervention types included in the analysis, even where no empirical analyses of these pairings are included in the review [1–3]. For example, a review of three interventions A, B and C which included empirical comparisons of all three against placebo, as well as A vs. C (but not A vs. B or B vs. C), could generate estimates of the effects of each pairwise contrast between A, B and C and against placebo. Multiple interventions meta-analysis may also be of particular use when included interventions are not only heterogeneous, but complex in nature [4, 5]. Here, we define ‘complex interventions’ as arising from combinations of components that interact with each other [6] and that operate through a variety of causal pathways [7, 8].

Also known as network meta-analysis, mixed treatment comparison and multiple treatments meta-analysis, the benefits of using multiple interventions meta-analysis (the term we use throughout this discussion) are several: it allows for the inclusion of all relevant evidence, including trials testing one active intervention against another and each relevant arm in trials with multiple arms; it allows head-to-head comparisons of distinct interventions; and when implemented with a Markov chain Monte Carlo method, it allows interventions to be ranked probabilistically by their relative efficacy [9, 10]. These methods are gaining increasing popularity and have been suggested for use with complex interventions [11]. However, they are not necessarily new. The first appearance of these methods for informing meta-analyses was to strengthen comparisons between two active interventions where the ‘direct’ trial evidence comparing the two interventions head-to-head was sparse [12]. Subsequent work focused on developing the statistical properties of this method [2, 13], including more recently by developing a comprehensive generalised linear modelling framework for its implementation [14]. Yet in all of this work, multiple interventions meta-analysis was primarily considered for use with pharmacological, ‘non-complex’ interventions.

Compared against reviews implementing pairwise meta-analyses synthesising evidence on one intervention or class of interventions against a comparison, multiple interventions meta-analyses present a fuller picture of the evidence than either ‘lumping’ analyses, which may mask important and clinically relevant heterogeneity across intervention types, or subgroup analyses by intervention type, which may be underpowered and difficult to hypothesise *a priori*. In particular, multiple interventions meta-analyses may be helpful both when a broad class of interventions is synthesised and when broadly similar interventions have different combinations of components. In this discussion, we specifically focus on heterogeneity arising from differences between interventions.

Yet despite suggestions of the suitability of multiple interventions meta-analysis for this task, and an existing literature describing the multiple sources of complexity and heterogeneity in systematic reviews of complex interventions [5, 15, 16], little attention has been given as to how to model this complexity and heterogeneity in multiple intervention meta-analyses. The goal of this discussion is to offer two specific approaches for engaging with complexity and heterogeneity in multiple interventions meta-analyses. As will be seen, these approaches have specific implications for the questions the meta-analyses inform and for the interpretation of these meta-analyses that are specific to complex interventions. Where this discussion goes beyond previous treatments of this topic [5, 11, 17] is in offering a specific taxonomy of approaches, with attendant implications for modelling complexity and for development of systematic review methodology. Here, we suggest two key approaches—the clinically meaningful unit and the use of components and dismantling—by which to understand the use of multiple interventions meta-analysis for heterogeneous groups of complex interventions. In ‘clinically meaningful units’ , interventions are grouped by similarity of modality or theory of change, whereas in ‘components and dismantling’ , key components or aspects of interventions are identified across included interventions, and labelling of interventions with these components informs subsequent meta-analyses. In practice, these two methods answer different questions. The use of clinically meaningful units helps in understanding which general approach is most effective, whereas the use of components and dismantling helps in understanding which components or combinations of components are associated with intervention effectiveness. Both of these methods are reflected in the way the interventions tested in included trials are sorted and parsed to form a network of evidence. A network of evidence represents the ‘mapping’ of direct comparisons between interventions (and combinations of components in interventions) as tested in included trials [10].

## Discussion

### Clinically meaningful units

Interventions that are labelled using consistent terminology from clinical practice, or which follow a generally understood and nominally distinguishable theory of change, may be grouped together into clinically meaningful units in a network of trials. In psychological and behavioural therapies, these may be known as ‘treatment modalities’—for example, cognitive behavioural therapy, motivational interviewing, or social cognitive theory-based interventions for health promotion outcomes. The rationale behind grouping interventions into these clinically meaningful units could be to class interventions into groups that are relevant and meaningful from a theoretical or clinical standpoint. Put otherwise, interventions may be grouped according to name brands (e.g. in parenting, where Incredible Years [18] and Triple P [19] are both marketed interventions), collections of activities or collections of hypothesised change pathways which, together, may represent a set of identifiable options for clinical decision-makers. From a research perspective, these groupings may also be used to test specific theories of change against each other. Overall, multiple interventions meta-analyses conducted in this framework seek to determine both which intervention approach or modality is most effective in addressing the outcome of interest, as well as what the relative effectiveness is of different approaches or modalities as compared against each other.

For example, Barth and colleagues [20] conducted a multiple interventions meta-analysis of 198 randomised trials of psychotherapeutic interventions for depressive disorders. Using an *a priori* taxonomy developed through expert consultation, they classed psychotherapeutic interventions into seven groups of therapies (e.g. cognitive behavioural therapy, psychodynamic therapy, social skills training) and categorised control groups into usual care, waitlist and placebo. Though Barth and colleagues excluded trials testing pharmacological interventions, other multiple interventions meta-analyses including both drug therapies and psychosocial interventions have grouped psychosocial interventions included in their trials to synthesise both types of evidence alongside each other. In their analysis of interventions for social anxiety disorder, Mayo-Wilson and colleagues [21] classed psychological interventions into groups including, amongst others, cognitive behavioural interventions (separated by individual or group), psychodynamic interventions, social skills interventions, and self-help interventions (separated by availability of therapist support). They meta-analysed these interventions alongside pharmacological interventions including selective serotonin reuptake inhibitors and benzodiazepines.

#### Benefits and drawbacks

The use of clinically meaningful units in the construction of the network of evidence has clear parallels in the first applications of multiple interventions meta-analysis for the meta-analysis of trials of pharmacological interventions. This points to a specific benefit of this method for constructing the network of evidence. Networks of evidence constructed using clinically meaningful units may more helpfully inform clinical commissioning and decision-making processes than networks constructed using components and dismantling, described below.

But this approach may only generate interpretable and relevant evidence when the taxonomy used to group interventions is also interpretable and relevant. In situations where the body of intervention trials to be synthesised is diffuse and includes many interventions with non-specific theories of change, clinically meaningful units may not in fact be very meaningful. Moreover, grouping interventions according to a taxonomy may mask important and clinically relevant heterogeneity within different instantiations of what is nominally the same intervention. For example, within the clinically meaningful unit of cognitive behavioural therapies, trials may use different manuals, focus on different aspects of this psychotherapeutic modality (e.g. goal setting vs. behavioural activation), and exhibit different levels of therapist drift into other modalities [22, 23].

Finally, meta-analysts will need to be attuned to the possibility that the statistical reality of the distribution of effect sizes in pairwise comparisons in the network may not match up with the way in which interventions will have been classed—that is, there may be underlying differences within the trials that may suggest that groups of trials are better split into two nodes in the network of evidence. In Kriston and colleagues’ multiple interventions meta-analysis of psychotherapeutic treatments for acute depressive disorder, conflict between two large trials testing the combination of cognitive behavioural therapy and medication accounted for most of the heterogeneity in their network of evidence [24]. More broadly, ‘inconsistency’, or situations in which the indirect comparison (i.e. a comparison between treatment B and C informed by trials comparing B vs. A and C vs. A) does not match up with the direct comparison (i.e. a comparison between B and C informed by B vs. C trials) [25, 26], may be an issue in multiple interventions meta-analyses led by this approach. Inconsistency may arise when interventions combined in one node in the network of evidence are better described as two or more separate nodes, possibly because the classification scheme conflates two or more distinct modalities, or because of heterogeneity explainable by any number of effect modifiers that may be imbalanced across different arms in the network of evidence.

### Components and dismantling

Instead of classing interventions into broad groups, another approach is to view included interventions as the combinations of components they represent. In this method, interventions are labelled via a system of components and grouped according to the combination of components they exhibit. Components may be defined as activities, as in ‘practice elements’ that represent a specific strategy to induce change [27], or as components based on their theoretical function [28]. This is akin to treating the body of evidence as a set of ‘dismantling trials’ comparing different combinations of components against each other. Outcomes of this analysis include not only the relevant pairwise comparisons estimating the relative effectiveness and summary ranking of different combinations of components as tested in the included trials, but also potentially the effectiveness of single components and interactions of components. Whereas the interpretation of a synthesis conducted using the clinically meaningful units approach is as if the analysis represents a large, synthetic *post hoc* multi-arm trial, the interpretation of a synthesis guided by components and dismantling is approximately like a synthetic factorial trial. That is to say, multiple interventions meta-analyses conducted in this framework seek to understand which components, or combinations of components, are associated with intervention effectiveness.

#### Combinations of components as clinically meaningful units

One approach is to treat each combination of components as their own ‘class’ of interventions. Put otherwise, each different combination of components manifested in included trials is treated as its own clinically meaningful unit. In a multiple interventions meta-analysis of interventions to promote uptake of smoke alarms, Cooper and colleagues [29] compared interventions including combinations of education with one or more of smoke alarm provision, fitting of smoke alarms and home inspection against usual care. Each combination of components present in included trials formed its own node in the network of evidence.

#### Summative component and interactive component models

Another approach using combinations of components is, as Welton and colleagues [30] propose, to treat the multiple interventions meta-analysis as a meta-regression where individual components are entered as meta-regressors. In fact, summative and interactive component models are conceptually equivalent between meta-regression and multiple interventions meta-analysis, though multiple interventions meta-analysis offers additional benefits in terms of flexibility of the modelling approach. Unlike the example above, this analysis represents a more substantial departure from the clinically meaningful units approach. In their multiple interventions meta-analysis of psychological interventions for clinical and psychological outcomes in coronary heart disease, Welton and colleagues [30] labelled each intervention according to whether they included one or more components from a prespecified list including cognitive therapies, educational activities, behavioural change, psychosocial support, and relaxation training. A set of models were hierarchically tested including a summative component model, where the effects of components were assumed to sum across interventions, and models with successively higher interaction effects between pairs and triplets of components—here called interactive component models. A more recent iteration of this approach was offered by Madan and colleagues in a multiple interventions meta-analysis of smoking cessation interventions [31]. In their analysis, they categorised interventions by the types of electronic or non-electronic interventions the interventions included and then tested interaction between electronic and non-electronic components, additive effects between electronic and non-electronic components, and whether electronic components could be considered equivalent in their effectiveness. It should be noted that when complex interventions include components that are hypothesised to work synergistically, interactive models are likely to be of theoretical importance.

#### Benefits and drawbacks

Both uses of the components and dismantling method model the heterogeneity present in included interventions, but in different ways. Treating combinations of components as clinically meaningful units encompasses the complex intersections, synergies and conflicts between components, though it misses the opportunity to specifically model these interaction effects. On the other hand, using summative component and interactive component models can to a degree test for the presence of these intersections. However, higher-order interactions between components (e.g. in Welton and colleagues’ [30] example above, four-way interactions) may not be included in the final model for several reasons—for example, the tradeoff between complexity and model fit indices, or inadequate data to inform specific interactions. In this case, the model assumes that these higher-order interactions are not in fact present (i.e. that components that are not ‘interacted’ in the model are additive), when these interactions would be detected with additional data.

On the whole, the use of components and dismantling can bring order to a heterogeneous set of interventions that may be poorly theorised, particularly when interventions are not readily classifiable by modality or theory of change. However, this benefit may be tempered by a lack of clarity as to what combinations of components actually represent beyond the abstract. For example, what shape should an intervention for coronary heart disease including cognitive and relaxation components take, especially if no single intervention included in the analysis included just these components, and if an intervention ‘combining’ these two components from across interventions that included these has not been specifically tested? This question is especially salient for future intervention design that seeks to optimise the use of effective components, such as in multiphase optimisation strategies [32]. There may be substantial clinical and programmatic heterogeneity that remains undertheorised and untested within specific combinations of components. Yet labelling components ‘transtheoretically’ across interventions with nominally different modalities may better account for heterogeneity in effect sizes than comparatively minor differences in intervention philosophy.

### Implications for research

Though the standard assumptions concerning multiple interventions meta-analysis apply in this context (for a full discussion, see [1]), meta-analysis of multiple complex heterogeneous interventions may require additional assumptions. For example, when using the clinically meaningful unit method, it is assumed that interventions in each unit are similar enough in theory and in delivery to be coherent. When using components and dismantling for key activities or change processes, it is assumed that, to approximate causal inference, the components scheme is exhaustive and covers all key active ingredients in included interventions, and that each component is coherent in terms of the activities or theoretically informed functions it contains. Otherwise, the results of the meta-analysis may inadequately explain heterogeneity in the included trials due to confounding by the differential presence of undetected or unlabelled components in certain component combinations instead of others, or the presence of effect modifiers that could meaningfully explain heterogeneity. We address both of these issues below.

First, it is important to consider the utility of these different approaches in ‘explaining’ (or, more accurately, exploring) heterogeneity in included trials, and to counterbalance this against the specific question that the meta-analysis seeks to inform. As highlighted above, a clinically meaningful unit approach may generate findings that more closely approximate the decisions that policymakers will take between different interventions. However, a multiple interventions meta-analysis conducted in this framework may mask substantial heterogeneity within relative treatment effects that may make intervention comparisons unhelpful due to imprecision. These meta-analyses may also combine interventions that are, in practice, implemented in a variety of ways. This heterogeneity may be better explained under an alternative classification method. In contrast, a components-based model may more adequately explain heterogeneity in the included interventions, but may say little about the interventional mechanisms at play and may thus be of lesser utility.

This tension between these two approaches goes beyond the different questions they seek to answer and speaks to the nature of how they explain the causal effects of these interventions. On the one hand, use of a theory-based classification method helps us to understand the ‘why’ of how interventions work by implying a set of causal mechanisms attributable to an intervention effect. By extension, a multiple interventions meta-analysis allows us to explore and compare different bundles of causal mechanisms to see which one is most effective. However, a theory-based classification method may suggest an incorrect or inadequate configuration of the evidence and may thus generate unhelpful results. On the other hand, use of a components and dismantling method (either as combinations of components or as summative and interactive component models) may better approximate what is being done in interventions, rather than the ‘why’ of how they work. This sacrifices the explanatory power of a theory-led approach for a potentially better-fitting model that accounts for greater amounts of heterogeneity. Another way of understanding this distinction is to consider it along lines of testing for ‘function’ and testing for ‘form’ across adaptations of complex interventions [28]—that is, in some cases, meta-analysts will be interested in testing hypothesised change mechanisms whereas in other cases, meta-analysts will be interested in testing specific activities as undertaken.

Second, it is possible that even when taxonomies of theories of change or intervention components are clear and exhaustive, substantial heterogeneity may remain due to other covariates. Meta-analysts should remain attuned to key moderators that may be associated with intervention effects, but that may not be directly captured by a classification scheme. For example, multiple interventions meta-analyses of pharmacological interventions have explored different doses of the same intervention and industry sponsorship of trials [33], and a variety of covariates relating to preparation and delivery of the intervention [34].

One common issue that systematic reviews of complex psychosocial interventions run into is a paucity, if not an absence, of high-quality evidence. This can lead to a situation where a pairwise intervention vs. control meta-analysis is unhelpful—perhaps when few trials are included, but each trial includes a categorically different intervention—but a multiple interventions meta-analysis either does not yield interpretable or reliable results or a network of evidence does not ‘connect’ due to inadequate data on different combinations. Madan and colleagues [31] highlight that this was a problem in considering the 36 different possible combinations of components in their multiple interventions meta-analysis of smoking cessation. By way of further example, in Freeman and colleagues’ [35] systematic review of behavioural interventions for childhood fecal incontinence and constipation, the ten included studies did not produce reliable results, and authors instead meta-analysed four studies comparing an intervention against treatment as usual.

Finally, several opportunities for further research present themselves. One key avenue is in comparing theory-led approaches to clinically meaningful units against component-based models to examine aspects of each that may be associated with improved ability to explain heterogeneity in included trials, especially when there is flexibility in the question the meta-analysis seeks to address, and in systematically examining the differences between meta-analyses using each approach. Another avenue is in the development of taxonomies of clinically meaningful units or components, and a related avenue is in methods for the development of these taxonomies in ways that are internally consistent within the systematic review. It is unclear what the basis of component labelling schemes should be, though one possibility is to use existing taxonomies of behaviour change, such as the Coventry, Aberdeen & London—Refined taxonomy [36], which offers a set of 26 possible behaviour change techniques. Methods for creating these taxonomies of components from a set of included interventions, such as Chorpita and colleagues’ [27, 37] approach to distillation of practice techniques, have been developed, though these approaches have largely been tested on empirically supported psychotherapies, especially for children. There is scope to develop ‘idiographic’ classifications specifically relevant to the interventions being synthesised using, for example, multi-method syntheses of programme-specific theories, process evaluations and other theoretical literature and interventional evidence, and then to use the results of these preceding syntheses to inform labelling of interventions. As a practical matter, two reviewers should independently undertake the labelling of components and clinically meaningful units, and the resultant configuration of the network of evidence, as is done for all other data extraction in systematic reviews.

## Summary

Multiple interventions meta-analysis is a method that holds promise for bringing increased relevance, utility and coherence to systematic reviews of heterogeneous complex interventions. A variety of approaches, including clinically meaningful units and components and dismantling, exist for developing a network of evidence to model this heterogeneity, though there are key areas that require further methodological development. The choice of methods should be guided by the substantive question—whether one of intervention modalities or of intervention components—the meta-analysis seeks to inform.
